# Living with *my* diabetes – introducing eHealth into daily practices of patients with type 2 diabetes mellitus

**DOI:** 10.1177/20552076241257052

**Published:** 2024-08-14

**Authors:** Catharina M van Leersum, Kornelia E Konrad, Marloes Bults, Marjolein EM den Ouden

**Affiliations:** 1 Department of Technology, Policy, and Society, Faculty of Behavioural, Management, and Social Sciences, 3230 University of Twente, Enschede, The Netherlands; 2 Faculty of Humanities, 10198 Open Universiteit Nederland, Heerlen, The Netherlands; 3 Technology, Health & Care Research Group, 2975 Saxion University of Applied Sciences, Enschede, The Netherlands

**Keywords:** Type 2 diabetes mellitus, eHealth, self-management, social practice, self-tracking, citizen science

## Abstract

**Objective:**

Diabetes patients can draw on an increasing number of eHealth apps to support them in the self-management of their disease. While studies so far have focused on patients with type 1 diabetes, we explored how patients with type 2 diabetes mellitus (T2DM) integrate eHealth apps into their practices aimed at managing and coping with the disease, which aspects were considered particularly valuable and which challenges users encountered.

**Methods:**

Semi-structured interviews and focus group sessions were conducted to explore how patients cope with T2DM in their daily lives and their attitude towards eHealth. In a further step, four eHealth apps were tested by patients and their expectations and experiences studied by way of qualitative interviews and focus groups.

**Results:**

The analysis showed that the study participants valued in particular the possibility to use eHealth apps to sense and gain a better understanding of their own body, to learn about specific responses of their body to nutrition and physical activity, and to support changes in daily routines and lifestyle. Key challenges encountered related to difficulties in interpreting the data, matching the data to other bodily sensations, getting overly occupied with the disease and difficulties in integrating the apps into personal, family, and care practices.

**Conclusion:**

Under certain conditions, eHealth can play an important role for patients in developing a nuanced, personal understanding of their body and coping with T2DM. A prerequisite is that eHealth needs to be fitted into the specific practices of users, and patients desire a strong role by their care professionals in providing support in interpretation of data.

## Introduction

Self-tracking practices have become increasingly common as a means to assist people in sensing and raising awareness of their bodies as well as changes within their bodies. Besides just feeling and sensing, it has been shown that users learn how their bodies react on behaviour such as physical activities, nutrition, sleep, and stress, which enables them to better diagnose and articulate their health status.^1^ On the other hand, critical perspectives on self-tracking have highlighted that self-tracking may also lead to people being overly preoccupied with their health status, or that visual and quantitative knowledge about one's body and health is privileged over other forms of self-perception, and collecting and making sense of data may turn into a burden in daily life.^2^

Patients with type 2 diabetes mellitus (T2DM) who regularly suffer from a high blood glucose level are a social group for that self-tracking may be particularly relevant.^3^ The patients do not always manage their disease properly, therefore, self-tracking could be beneficial for these patients, because controlling the disease requires self-management and reacting on changing personal health status over time.^4^ Besides a genetic predisposition, patients with T2DM often have unhealthy behaviours that are challenging to change.^5^ Thus, to address such challenges, the self-management activities could entail healthy nutrition, physical activity, monitoring of blood glucose levels, adherence to medication, and routine medical checks.^4,6^ Self-tracking could enhance someone's knowledge about their diabetes and provide motivation to work towards a healthy lifestyle.^7^ Electronic health technology (eHealth) could be used to track and store health data. It has also been suggested that self-tracked data could serve as an objective and reliable additional source of knowledge providing insight into normally invisible health aspects.^8^ However, patients with type 2 diabetes mellitus (T2DM) perceive several challenges in translating the self-tracked data into coping strategies.^9^ The challenges occur due to an overload of data and information or conflicting information, which requires the need for easy access, tailored information suiting individual needs, and support of care professionals.^9^

Earlier research by Danesi et al. investigated how glucose monitoring technology was used by adolescents with type 1 diabetes mellitus (T1DM).^10^ Their study showed mixed experiences by the users. Part of the users experienced the monitoring technology as helpful for becoming more responsible in understanding their disease as well as self-manage a healthy lifestyle.^10^The patients developed an increased self-awareness and understanding of the relation between treatments, nutrition, and physical activities. However, Danesi et al. also showed that some patients and care professionals considered the device as less helpful as the multitude of generated data was difficult to interpret or considered imprecise, and other users abandoned the device for various reasons.^10^ Furthermore, they point towards the crucial role of care professionals in providing information and support for using eHealth.

Thus, former research indicates that eHealth could constitute a valuable element to enhance T2DM patients’ self-management practices, but it is not clear to what extent experiences gained by patients with T1DM who are typically of a different age group and with a different disease expression are similar to those of T2DM patients. While the majority of studies on eHealth addresses T1DM, T2DM is a much more common health condition. Furthermore, the perspectives and experiences of this user group might also be different compared to the often health and fitness oriented groups that have been largely the focus of self-tracking research so far.^2,10^

In order to explore how T2DM patients integrate eHealth apps in their personal, family, and care practices, this study has been informed by a social practice perspective. The social practices approach focuses on recurring and meaningful activities of people in their daily or professional life, by conceptualizing common and routinized ways of performing something as a practice.^11^ A practice can be defined as a behaviour influenced by and connected with bodily activities, mental activities, the use of technologies, understandings, emotions, and motivation.^12^ As indicated by the very notion of ‘social practices’, the social practice approach pays attention to the way practices are shared among social groups, the way they are embedded in or connected with other practices and the practices of others. An important element in a social practices analysis is how practices change. In this study, changes resulted after being diagnosed with T2DM and after starting to use eHealth to support self-management. The social practice approach is particularly suitable for tracing how people integrate new devices and data into their everyday life, how these new elements are related to their established activities, knowledge, skills, understandings, values, as well as networks of related practices of themselves and others in their personal and care networks. This is important for understanding how eHealth is used by patients with T2DM, how it matters for self-management practices, personal and care relations, or how other less obviously related practices may be affected.^13^

The aim of our study was to learn about the possible roles of eHealth apps in supporting patients with T2DM and their user experiences in daily practice. In particular, we ask how T2DM patients integrate eHealth apps into their personal, family, and care practices aimed at managing and coping with the disease, which aspects were considered particularly valuable and which challenges were encountered. We chose for a qualitative study approach informed by a social practice perspective, in order to get detailed insight into the lived experience of patients with T2DM. This is important to develop an understanding of the particular ways how eHealth apps are used, how users generate use value for themselves, and what problems underlie the challenges users may encounter. In our study, we first conducted interviews and focus groups with T2DM patients to learn about their practices in coping with diabetes in their daily lives. This step aimed to gain a general understanding of coping strategies of T2DM patients irrespective whether they used eHealth apps or not. In a further step, four different apps with different functionalities were offered to patients with T2DM, to investigate the daily self-management practices with eHealth and the added values of eHealth in more depth.

The article is organized as follows: First we review insight on the relation between T2DM, self-management, and the role of eHealth, and motivate the choice of eHealth applications used in our study. This is followed by a presentation of our study and our findings, starting with the sometimes more, sometimes less successful integration of eHealth into patients’ practices of sensing their bodies and of understanding how *their* body relates to nutrition and physical exercise. Then we broaden the perspective beyond the individuals to considerations on how accessibility of eHealth apps is mediated by the health system and, finally, how the networks of relatives, friends, and care professionals are involved in and affected by self-management and self-tracking practices.

### Background on eHealth and self-management of diabetes mellitus

Diabetes has two primary types. T1DM is an autoimmune disorder and affects approximately 9% of patients. T2DM includes impaired insulin secretion or a resistance of the body to insulin, usually diagnosed in adults of 40 years and older.^14^ Just as the study of Danesi et al., most research on eHealth focussed on T1DM and not on patients with T2DM.^10,15–17,4^ However, T2DM is the most common type of diabetes and an increasingly prevalent disease.^5,11^ In contrast to T1DM, T2DM can be promoted by physical inactivity, overweight, and nutrition,^18,19^ which could be overcome by weight loss and improvement of metabolic parameters.^20^ Patients with T2DM can even end in remission when they adhere to rigid self-management practices.^21^ Based on the expectation that eHealth enables better self-management of health conditions, governments and some healthcare organizations invest more and more in the use of eHealth.^22,23^ However, patients do not often use eHealth to support them and professionals do not often recommend eHealth use.^7,21,23^

Gimbel et al. showed that current T2DM treatments and lifestyle interventions are insufficient to improve self-management.^24^ Most patients with T2DM face the challenge to continuously adjust their drug treatment in order to live well.^25^ Besides the possibility to treat with medication, the support of a healthy lifestyle including education on lifestyle for diabetes self-management can assist patients with the control of their blood glucose levels and lower the progression of diabetes.^10^ Studies have shown that being physically active and being aware of nutrition intake leading to weight loss is effective to improve metabolic parameters of patients with T2DM,^20^ and remission of T2DM.^21^ Changing lifestyle could potentially decrease the need for medication and prevent or delay T2DM and possible side-effects.^26,27^

To assist and improve self-management practices, web or app-based eHealth tools have shown to be beneficial in improving a healthy lifestyle,^7,28^ especially if the apps contain an integrated personalized feedback system.^4^ Different apps have shown promising effects on the treatment as well as prevention of T2DM.^29^ eHealth can provide T2DM patients access to real life data that assist in changing daily routines^30^ or connect with healthcare professionals.^31^ Especially this connection with professionals appears to be important for patients with T2DM,^32,33^ and lifestyle coaching combined with eHealth seems promising.^34^ To offer this combination, care professionals (e.g., general practitioners, nurses, and dietitians) need to be familiar with eHealth tools, their uses and potential, in order to consider whether such an app may assist in someone's lifestyle.^30^ However, due to the extensive offer of apps with diverse functionalities it is hard to judge which are reliable, and the information given to patients through the apps is questioned by care professionals,^35^ and their recommendations are limited to a small number of apps, limiting the free choice of T2DM patients.^36^ The care professional consulted by patients with T2DM can give a referral to a dietician to receive assistance with their nutrition or weight management, but the use of apps or eHealth is not included in current T2DM care protocols.^37^

Although eHealth is not taken into protocols, some healthcare professionals recommend the use to their patients. They are likely to recommend if the app is easy to use and access, provide real-time feedback about nutrition, activity, or blood glucose, is free of charge, and gives the professionals the possibility to communicate with the patient.^38^ However, there is also hesitation because most consider the use of these technologies as too difficult for patients with T2DM. Furthermore, professionals may not recommend the use of eHealth since they themselves are often unfamiliar with the existence of the self-management apps, and there is much unknown about costs, regulations, and how to bill this kind of online care.^33,39^

### Choice of eHealth apps covered in our study

Previous research has investigated one eHealth tool as part of one study or taken eHealth in general.^10,15–17^ Since there is a multiplicity of apps with very different functionalities available, we included multiple apps covering a range of possible functionalities to develop a broadly based understanding of how users incorporate them in their self-management practices and how they experience the value of the apps. The choice of apps to be included in our study was guided by former research indicating which types of apps are most interesting to T2DM patients. In an earlier study, we collected quantitative data from patients with T2DM.^40^ The most frequently mentioned technologies or apps were the diabetes lancing devices, online diaries, and step counters. None of the apps they used had the ability to collect health data, such as blood glucose levels. According to a systematic review of Alaslawi et al., patients with T2DM are most interested in functions considering nutrition or diets, such as diet tracking, calorie counting, and recipes.^32^ Second favoured is the possibility to monitor blood glucose level with diaries and reminders to check the levels on a regular basis. Other desired functions were activity trackers, step counters, and reminders to support physical activity.^32^ Based on these preferred function, a long list with apps was made, and four apps were chosen which cover the known spectrum of self-management practices for patients with T2DM. Furthermore, the companies of these apps were enthusiastic to collaborate in this study.

Thus, we offered four different apps to patients with T2DM, including Clear, MiGuide, Selfcare, and mySugr.^41^ The Clear app used the FreeStyle Libre continuous blood glucose monitoring system to provide insight into users's response to nutrition and physical activity, and the user can register stress levels and sleep patterns. MiGuide is designed to provide blended care by focussing on someone's lifestyle and behaviour and coaching on the basis of food intake and daily activities. Selfcare is an app in which users can monitor their personal health values, such as blood glucose level, weight, heart rate, and blood pressure, by connecting to different sensors or wearables. This app included a platform with challenge-based gamification. The mySugr app can be used to register blood glucose levels, food, and medication.

## Method

### Setting and recruitment

This study is part of TOPFIT Citizenlab,^1^ which is a research programme in the Netherlands aiming to collaborate with citizens to investigate their health needs and practices, how they cope with their disease, and explore the potential of digital technology for creating personalized care services. The Citizenlab researchers apply citizen science methods to design the studies, collect data, and perform the analysis, and to reach active collaboration with different stakeholders.^42^ In this study, we collaborated closely with patients with T2DM and developers of the four eHealth apps. We chose for a combination of methods to increase the credibility^43^ and ensure to understand a broad variety of self-management practices of patients with T2DM. The data collection took place at the University of Twente in the Netherlands as well as online and via phone calls. All participants lived in the Netherlands

Our research consisted of three data collection steps. First, we conducted interviews to understand how patients cope with T2DM, and their attitude towards eHealth. Second, we organized focus groups to discuss the tasks, roles, responsibilities, and struggles of living with T2DM in more detail. And third, T2DM patients tested the four different apps to explore expectations and experiences with eHealth. Convenience sampling was used to recruit participants. The inclusion criteria were: having a diagnosed T2DM (any disease duration was included), aged ≥18 years, and living in the Netherlands. We recruited the participants via the Dutch Diabetes Association, flyers, and announcements on social and regional media. All those interested in participation received follow-up information and were contacted by the researchers. Those who were willing to participate received the informed consent form. All people willing to participate were allowed to join in this research, none had to be excluded. The same recruitment strategy was used for each data collection step, leading into three different groups of participants. Ethical approval for this research was obtained from the Ethics Committee of the University of Twente (reference numbers 201213 and 210043). All participants were informed about the study and their right to withdraw at any time. Data were anonymized, and confidentiality was maintained.

### Data collection

When someone receives the diagnosis T2DM, they have to adapt their life and cope with this new diagnosis. The current study was applied to explore the subjective experiences and self-management practices of patients with T2DM, and their expectations and experiences regarding different forms of eHealth.^43^ First, a qualitative descriptive research design was chosen to delve into the lives of patients with T2DM and their attitude towards eHealth. The data collection consisted of semi-structured phone interviews which explored how each individual cope with T2DM. The interview guide was designed on the basis of findings from earlier research.^40^ We conducted 16 phone interviews with patients with T2DM of which six did not use technologies and 10 used technologies to self-manage their diabetes (Table 1). The phone interviews lasted 30 to 60 min and were conducted in October and November 2020 by four researchers. The interviews were audio recorded, field notes were written, and verbatim transcripts were made of the audio files (see interview guide in the appendix 1). As a member check,^43^ all participants received a summary with an infographic based on the results. The goal of these more exploratory interviews was to get a first understanding how patients cope with T2DM in their daily life, and their stance towards eHealth.

**Table 1.** The different data collection methods of this study with the number of participants and participant characteristics.

	First data collection	Second data collection	Third data collection
Methods	Individual interviews	Four online focus groups	Individual interview before use Test period of eHealth Focus group after use
Number of participants	16	8	25
Gender			
Male	10	7	12
Female	6	1	13
Other	0	0	0
Age			
30–49	2	0	1
50–69	7	4	18
≥ 70	7	4	6
Nationality	All Dutch	All Dutch	23 Dutch and 2 other
Disease duration			
1–4 years	0	0	4
5–9 yeas	3	2	7
≥ 10 years	13	6	14
Use of technology before	10	6	13

Patients with T2DM have different tasks, roles, responsibilities, and struggles in their daily practices. The second data collection consisted of four online focus groups that allowed us to gain a better understanding of these aspects, as well as the usability of eHealth apps they were using. The sessions were organized with eight patients with T2DM of which two had never used technologies and six used technologies to cope with T2DM (Table 1). The goal was to share ideas and different experiences in living with T2DM, and to discuss and formulate desires and ideas for assistance and improvements of diabetes care with eHealth. The focus groups had a co-creation format in which the participants shared experiences in coping with their disease, decided on mutual challenges, and designed research or took actions to overcome these challenges. The study tools of the first session were created by the researchers and consisted of three themes to discuss: tasks when having T2DM, roles and responsibilities with T2DM, and struggles in daily practice with T2DM. At the end of each session, the structure of the next session was designed and discussed with all participants. The sessions were conducted between December 2020 and June 2021. Each session was a continuation of the previous session, they all lasted for 90 min with the same participant group. During the online session, three researchers were present. One researcher had the lead, one was present to assist the participants with all technical difficulties, and one researcher made observations and field notes. An extensive summary was made based on the field notes, which was directly distributed among all participants. With this member check,^43^ the participants had the possibility to react and elaborate on the topic as well as use the document as a preparation for the next session.

The focus groups, of the second step of data collection, revealed a variety of desires in coping with T2DM and the participants expected that eHealth could contribute to these desires. This finding was applied in the following research step, the third step of data collection that was designed to actually test the use of the different eHealth apps in practice with a larger group of participants. Twenty-five patients with T2DM were recruited to test one of the chosen eHealth apps during a period of four months (Table 1). The participants were only included if they did not have experience with the particular apps before the study. This third step consisted of interviews before the use of the chosen app, asking patients about their self-management practices and expectations of eHealth, then an individual test period of four months, and final focus groups to discuss experiences with the app, how it contributed or changed their self-management practices, and to what extent it is or could be used as part of their daily life. The interview guide was made on the basis of findings of the earlier steps, and in consultation with the participants of the second data collection step. The focus group topic list was created in collaboration with two participants who were testing the apps (see interview guide and focus group topic list in the appendix 2 and 3). All participants met with the app developers during a webinar, after which they could choose which app they wanted to test. Twelve participants chose to start using Clear, five Miguide, four Selfcare, and four mySugr. Most were eager to test the Clear app due to its functionality of combining continuous blood glucose monitoring with nutrition and physical activity practices. However, not all wanted to have continuous blood glucose monitoring, but if they wanted to include blood glucose values their choice was the mySugr app. For those who did not want to have any glucose monitoring, the MiGuide app preferred above the Selfcare. Although both offer a broad range of health value tracking options, MiGuide had specific functionalities for patient with diabetes. Phone interviews with the participants were conducted between April and May 2021, and lasted for 30 to 60 min. In September 2021, four focus groups were organized at the University of Twente. Two focus groups with users of Clear (12 users), one with users of MiGuide (five user) and one with users of Selfcare and mySugr (one Selfcare and two mySugr users). The two participants who collaborated to create the topic list as well volunteered to lead the focus groups. This focus group format was chosen to create an environment in which the participants could share experiences and learn from each other. The sessions lasted 90 min, audio-recordings and field notes were taken, and an extensive summary was made and shared with all participants in order to give them the opportunity to read and react.^43^

Overall, the participants in the different steps of the study covered rather well male and female users, and the main age groups of T2DM patients. However, participants show a bias towards Dutch participants and having been diagnosed with diabetes for some time already. This can be attributed to the recruitment strategy. The latter implies that participants have most likely already established personal practices of coping with T2DM, which can be perceived as valuable for our study. The small number of non-Dutch participants, however, implies that the results of our study may not be fully transferable to other cultural groups that could differ with regard to health conceptions, practices, and use of digital technologies.^44^

### Data analysis

We applied a content analysis to all data obtained during the interviews and focus groups. First, inductive coding was applied to observe and define different themes.^45^ We read the transcripts and codes were accorded to the quotes. Second, all codes emerging from the data were combined into a list of overarching themes. Finally, two researchers discussed the overarching themes, after which one researcher coded all files and discussed findings with the research team at weekly meetings. All participants spoke the Dutch language, therefore, the transcripts were all in Dutch and English translations were made of the quotes used in the results section by the authors. The researchers used software package NVivo 11. In each data collection step, data saturation was reached when no new themes emerged.

### Trustworthiness

Credibility was established through several procedures.^43^ We combined interviews and focus groups (method triangulation) with audio-recordings, transcripts, and observations with extensive notes to establish the credibility of the data and support the findings with quotes of different participants. Several researchers collaborated (investigator triangulation) in the design, execution, and analysis of the data. To align the research methods, we designed and discussed our study tools thoroughly during weekly meetings. During these meetings, the experiences of conducted interviews were shared to create alignment among the researchers (peer debriefing). Furthermore, the research team consisted of professional researchers and patients with T2DM, and summaries and infographics of preliminary findings were shared with all participants (member check).

## Results

**Figure 1.**
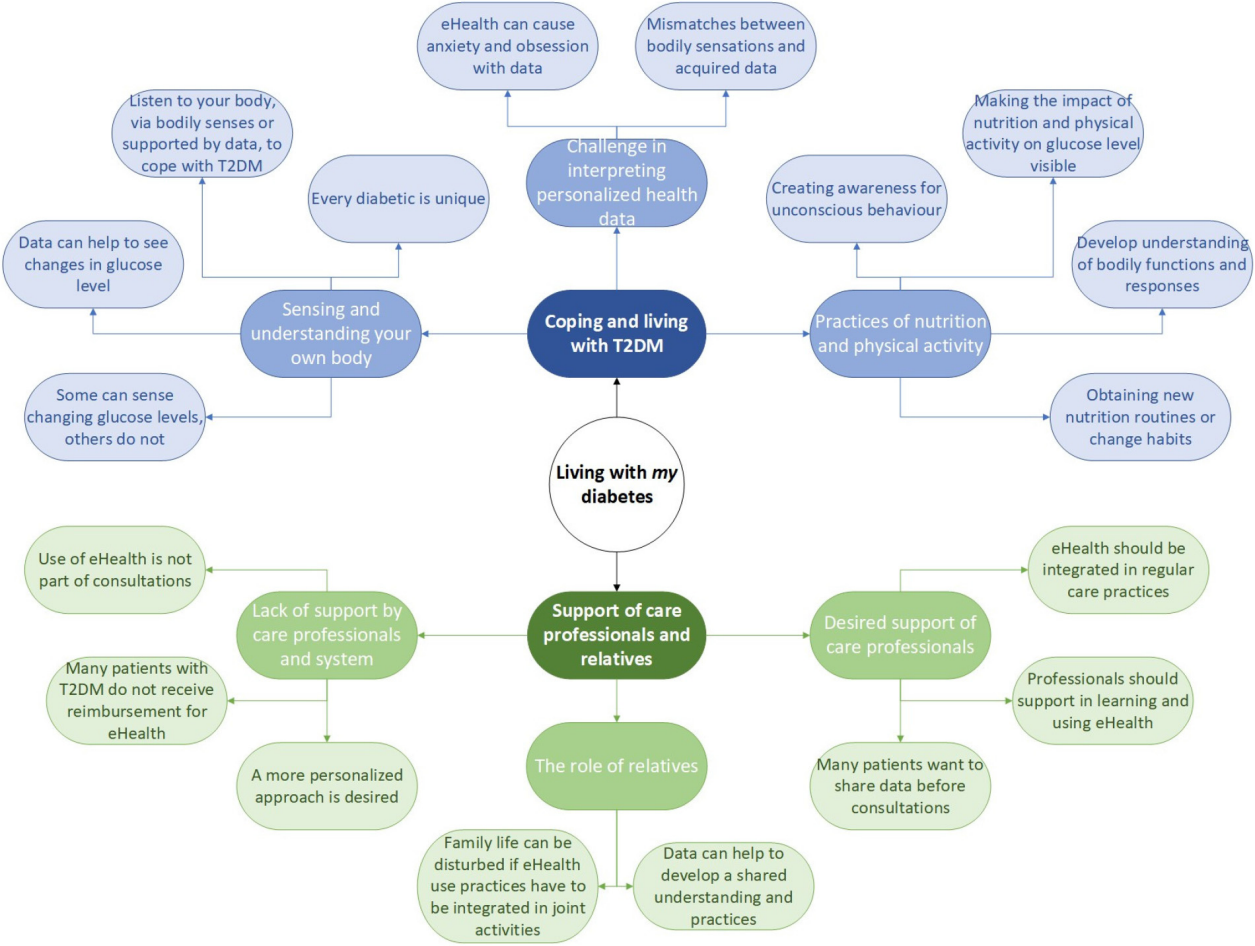
Summarized representation of main findings for each sub theme.

The data analysis revealed (1) how patients diagnosed with T2DM get grips on their life and understand their body with or without eHealth and (2) the role of support of care professionals and relatives. Each of these topics is addressed in the following. The main findings are summarized in figure 1.

### Coping and living with T2DM

The diagnosis T2DM has a great impact on the lives of people. How people change practices due to diabetes, how they always need to be aware of their illness, and how they understand their own body and its specific reactions were important topics related to the first theme: coping and living with T2DM. This first theme will be divided in three sections including practices of sensing your body, challenges in using personalized health data, and adjusting practices related to nutrition and physical activities.

### Sensing and understanding your own body

Coping with diabetes is a learning process, not the least because patients must learn to understand and listen to their own body. Most participants said that they got used to this and know or feel if their glucose level changes, especially when the level drastically increases or decreases. ’*I also suffer from neuropathy, therefore, I notice an increasing glucose level in my toes. Listening to my body helps in coping with the diabetes.’* However, some argued that this is not always easy, leading to unfortunate events when glucose levels drastically decrease and they collapse. A prerequisite, before being able to listen to their body and know how to cope with the signals, is that patients with T2DM need to have a certain awareness of their own body and its deficits. Another topic considered the influence of medication and insulin, all participants used medication to control their disease. Participants had a strong desire to decrease the amount of medication or if possible live medication-free. Earlier research showed that medication has an influence on bodily sensations.^46^ Our participants agreed that medication helps to lower glucose levels, but at the same time makes it more difficult to be aware of the bodily sensations experienced by T2DM.

eHealth could provide a way to sense your own body. Less than half of our participants, who were interviewed in the first study, used eHealth to track any health related values. Those who used apps to monitor their health saw the app as an external device which helps you to ‘*forget diabetes occasionally and get used to a necessary new behaviour.’* One sophisticated form is sensing through (continuous) monitoring. Several apps provide real-life data, and Clear, for example, provides continuous blood glucose level monitoring. Although some patients with T2DM argued that they feel changes in their body and know when to eat something, others do not recognize symptoms before a dangerous low blood glucose level (hypo). One participant referred to an unexpected and first experience with hypo which lead into a critical situation because he fell from the stairs and his wife had to call an ambulance. After this first experience he had more hypos, but still did not recognize any bodily sensation. The data from the mySugr app helped him a lot to see the changes in his blood glucose level. Liu et al. argue that these measurements compute and detect worsening symptoms, which is important to provide available data that can help patients to react when needed.^19^ This was what exactly was expressed by our participants as well. They used the data from the app to see their personal health status. Furthermore, as a study by Zang et al. also shows,^47^ most of them already experienced a positive effect on their glucose level in the first months after using the apps.

Also our participants in our study argue that acquiring real-time data with the use of eHealth would be beneficial, both for patients who are recently diagnosed with diabetes and for those who are in a more advanced stage. The recently diagnosed patients could become able to understand the influence of nutrition and physical activity in an early stage, act in this early stage, and prevent progression of the disease due to insight in their health data. Patients in a more advanced stage could use the data to gain deeper understanding of their current lifestyle, and try to reduce the use and need for medication. Reduction in medication need was experienced as a large benefit of using the apps, and some of our participants mentioned that they already spoke with their care professionals about a possible change in medication due to stabilized blood glucose levels after they have started using the apps. During one focus group session the conclusion was that eHealth is desired ‘*to access personal bodily, real-life data and support in changing daily routines and lifestyle, which could in turn prevent T2DM and lower the overall prevalence of T2DM.’* Lowering the prevalence of T2DM would not only be beneficial for the healthcare system, but also for society at large.

### Challenges in interpreting personalized health data

While participants generally agreed that the apps assisted in sensing and understanding their body, some patients struggled with mismatches between bodily sensations and data from the apps, or with data that were hard to interpret for them. As one participant experienced during the test of Clear: ‘*when I measure a high glucose level, and I do not feel different I consider the measurement as a fault.’* If there is a mismatch between someone's bodily sensation and the output they receive from the technology, the participants argue that they will not trust the technology and do not know how to interpret this output. Furthermore, participants acknowledged that the use of eHealth can negatively impact someone's daily life, especially if they do not know how to interpret the data. The data could provide a false confidence or provide a false notification that they are not doing well. An example given by one participant, who already used eHealth at the moment of the first interview, was of having an occasional ‘bad glucose level’ seemingly caused by eating a banana. On another day he again ate a banana, which did not cause a high glucose level. Since he knew a banana was healthy he did not worry, but gave the example to show how confusing the output could be. When the apps become part of someone's life and influence the decision-making it could lead towards dangerous situations when ‘wrong’ nutrition or actions are healthy according to the app. What becomes visible here is that the measurements are not considered in isolation, but in the context of further personal sensations and knowledge about oneself. Understanding of all aspects was needed, as the participants argued, to actually capture their life and have the possibility to change their lifestyle. Lupton and Milne et al. talk about a data double or digital twin to categorize and identify ‘at-risk’ situations, and feeding this data back to the user to encourage action.^2,48^ Some of our participants who tested apps in our study would prefer to have such a digital twin to increase guidance provided through the apps and make it more personalized as a kind of remedy for the limitations and blind spots in measurement.

Besides the sensation of a mismatch or confusion, being continuously occupied with an illness could also cause anxiety. This was confirmed by one of the participants of the focus groups ‘*with those technologies I want to know at any time how I am doing, it almost becomes an obsession.’* Due to eHealth this person was even more preoccupied with diabetes. The use of eHealth made him ‘act too much’ and measure the glucose levels more often than needed. He was already insecure about having diabetes and understanding the changing glucose levels, which is not the case of all patients with T2DM. Another participant experienced the anxiety as well when testing the Clear app. ‘*Using Clear made me nervous and insecure.’* Due to the insight obtained with the app, he became even more aware and concerned about his diabetes, and all information could feel overwhelming.^9^ His diabetic nurse advised to stop using the app. This was experienced as a pity by the participant, but at the same time as a relief and possibility to be less anxious about his illness and lifestyle. According to another participant of the focus groups, it is very important to understand the data obtained through eHealth and know how to react on obtained numbers. Low level of understanding could lead to anxiety and ‘acting too much’.

### Understanding the influence of practices of nutrition and physical activity on your own body

Confirming previous studies, such as Mayo and Nash,^49,50^ the diagnosis T2DM was a life-changing event for all participants in our research. All had their own story on how their life changed by the diagnosis. Most patients did not change their behaviour at the onset of T2DM, but soon realized that they needed to do so to decrease symptoms, such as a dry mouth and fatigue. One participant told about his diabetes-bag that he takes with him wherever he goes. Among others, this bag contains food, with medium-acting carbs and with fast-acting carbs, and a bottle of water. At first it was hard to cope with T2DM for him, understand the impact of diabetes on his body and include needed actions in his life, but later it became a routine element of his daily practices. He said: ‘*It is just part of my life, and I take the bag wherever I go.*’ All participants stated that they had to change their lives to cope with T2DM and take actions to include the changes in their new and healthy lifestyle. Amongst others, a change in lifestyle to lower glucose levels by changing nutrition and doing sports or other activities. Although patients can redesign their life, they are always confronted with their diabetes. ‘*You can never have a day off from diabetes.’* Another important observation remarked by multiple participants, that diabetes is different for every individual. ‘*Every diabetic is unique. Carrots are healthy for some, but for me they are the worst.’*

Similar to earlier studies, such as Danesi e al. or Lupton,^2,10^ our study shows that technology can play a role in learning and understanding the influence of nutrition or physical activity on the body. The participants who used Clear or mySugr and self-monitored their glucose levels strongly agreed with this statement. They stated that insight through digital technology is very valuable ‘*to understand and connect bodily sensations to actual numbers’* (i.e., glucose levels). For example, one participant knew that fries had a negative effect on the blood glucose level, but the enormous increase was not expected. The app increased awareness of this ‘unhealthy’ nutrition which led to a change in lifestyle according to our participant. The MiGuide app provided tips to change lifestyle. Some of these tips included physical exercises. After taking these tips into a daily habit the users saw stable data and experienced a feeling of healthiness. ‘*Having T2DM made me aware of the importance to structure my life more and especially physical activity. Since the use of MiGuide I also use a pedometer and I feel much better than before.’* Self-monitoring can raise awareness of their lifestyle, increase self-care and empowerment of patients with T2DM by access to, use of, and sharing personal data. Participants who tested the different apps experienced the use often as pleasant and motivating. Motivating in a sense to work on their lifestyle and change their lives because the app provides insight in you daily habits. ‘*Once you notice the results you will do even more healthy. It is a kind of making aware, because you are going to visualize everything and making unconscious behaviour visible. It helps a lot to not just eat everything.’*

Participants who tested the apps sometimes experienced difficulties in fitting the app in their nutritional practices, which is a prerequisite to ensure long-term use. For one participant the use of eHealth has to fit with his allergy to nuts. He tried an app with recipes for patients with T2DM, but for him this was not an option, because it was not possible in the app to receive recipes without nuts. Although the app Clear gave insight in glucose levels and assisted in changing nutrition, some participants argued that the use of the app did not always fit with the practices of the user. ‘*I am not eager to weigh all my food and note this in the app. Then a meal will cost half an hour extra just to search and use the app. If this is needed, I am happy the way I am now.’* The apps mySugr and Clear assisted in understanding the effect of certain nutrition on glucose levels and when to avoid or change meals, which was the knowledge most participants liked to obtain. The participants who tested the MiGuide or Selfcare app as well acknowledged that they would see this as a needed adjustment of the apps.

All participants agreed that eHealth provided essential insight to assist in changing their lifestyle. It could enhance their abilities to provide self-care and cope with T2DM.^51^ In particular, participants were interested in obtaining personal body data and getting access to information on their own unique expression of diabetes. This type of personal health knowledge differs fundamentally from knowledge and advice based on generalized insight based on regular health research. As explained by one participant, this type of personal health data can give the required insight to adjust their lifestyle. This person was able to see the changes in their glucose levels and could try to lower these levels with different nutrition and physical activities. ‘*Understanding the relation of nutrition or physical activity on my glucose level, and being able to see the changing glucose levels made it so much easier to cope with my diabetes.’* By using the Selfcare app she gained insight in data about among others blood glucose level, weight, and heart rate, which assisted in exploring the effect of nutrition and physical activity and assisted her to choose foods with a low influence on her ‘normal’ data. Access to real-time data increased the perception of T2DM and helped to change practices for many of our participants. The more generalized advice provided by some of the nutrition apps was, however, considered as too unspecific by some of the participants. ‘*When the app tells me to eat less carbohydrates, I know. It also gives examples like bread or pasta, but I would like to get direct feedback on the food I have been eating and how to improve.’* However, most patients with T2DM do not have access to their bodily data and the technology is not available for everyone. As stated by one participant during a focus group session, ‘*technology should be available for everyone’*, which should be made possible by our healthcare system.

### Support of care professionals and relatives

Patients with T2DM were in the focus of our study. Still, these patients are part of a larger network of relatives and friends, as well as a care network. Patients need support in their daily practices, knowledge, and understanding of data, and use of eHealth apps by these relevant others, and their actions may influence these others as well. In this section we first look into the role of relatives, then address the lack of support from health professionals experienced by some of our participants and, finally, the sort of support from healthcare professionals that is desired by our participants.

### The role of relatives

Besides patients with T2DM, their family and friends get involved in daily practices as well. In our study it was clear that most participants had a supporting network, but in some cases the relatives were not that supportive or concerned with the illness. Where almost all participants were supported by their partner, most were less supported or understood by friends, for instance when conflicting with established social practices. For example, when rejecting a piece of cake at a party this was questioned and not taken too serious. One participant told ‘*when I visit my friends or family, they always offer me a piece of pie, even though they know I don’t want it. For them it is a way of being polite, for me it was a hustle to always reject. Now after 15 years they know and have stopped asking if I would like a piece.’* One of our participants told about the challenges of Selfcare and how these were taken in the family. For example, there was a challenge to eat only whole-grain foods for an entire week. His wife and children enjoyed participating in this challenge and were eager to support. This already shows that the use of eHealth as well influenced the lives of the people in their network and was considered to improve support by their network.^52^

However, besides this enthusiastic family, there were other partners very positive about the ease of the eHealth and the value it has on living with someone with diabetes. ‘*My partner encouraged me to keep tracking all data. With the data we could decide on recipes most beneficial for my health.’* One participant told about the curiosity of his colleagues at work. They were asking him to show how the Clear app worked and they wanted to use the app as well to check his glucose level. Besides enthusiasm, there were partners less excited about the impact of eHealth. All four apps had an impact during the entire day and kept the participants occupied. The need to frequently use their mobile phone was not only disturbing for themselves, but also an annoying behaviour according to their network. It had a large influence on their daily practices shared with other family members, such as making photos of their meal and recording all calories or physical activity during the entire day. ‘*My wife asked me to stop using my phone during dinner.’*

### Lack of support by healthcare professionals and system

The monitoring devices used by our participants were designed for patients with T1DM. Some other participants already knew about these apps as well, but did not make use of the apps due to costs and lack of reimbursement by insurance companies for patients with T2DM. ‘*It is a pity, you want to do some extra things, measure and have more control over the disease, but if you do not use insulin you will not receive reimbursement. For me it is too expensive.’* They argue that the healthcare system and the professionals should support them and increase the possibility to use apps, because to understand the illness and understand how diabetes affects the individual, access to real-time data is desired by most. As part of the current healthcare system, all participants had regular consults at a general practice where their blood glucose level was measured, but these are just snapshots. ‘*I have to visit my general practitioner regularly to measure the glucose levels and blood pressure. They puncture my finger, but this is just a single moment.’* The treatment and advice patients receive is based on these snapshots and the average patient with T2DM. A hypo, for example, cannot be predicted based on these snapshots, and it is of clinical and personal interest to predict these events.

Similar to the findings of Danesi et al. on patients with T1DM, care professionals have a crucial role in the lives of patients with T2DM in understanding their illness or the data they might obtain by using eHealth.^10^ They argue that professionals can support in coping with diabetes, interpreting (measurements of) glucose levels, and acting on the data. The perceptions of the participant in our study on the role of care professionals is varied, not only about the intensity, but particularly about the type of support they would like to have. Some participants had a good relationship with their professional and others felt they were treated only according to protocol. A more personalized approach is desired by most participants, ‘*we are not just a number, and continuation or change of medication is the only thing they discuss with me.’* The lack of support in changing their lifestyle made them unable to abide to, for example, a weight loss goal, or did not give desired guidance in finding knowledge about healthy nutrition. Some mentioned that they received a referral to a dietician, but none was satisfied with the assistance of the dieticians. eHealth was considered a solution for them, but the use of technology was not part of the consults with most care professionals.

### Desired support of healthcare professionals

Since eHealth for patients with T2DM is available, all participants argued that it should be integrated in ‘regular’ care, and the professional is the first who should inform patients about eHealth. Most participants thought that they could benefit from the use of eHealth. Not only to improve their daily life with diabetes, but also to improve the patient–professional relationship because the available personal body data could enhance personalized care practices. ‘*It would be really good to share data on a regular basis or before a consult. Then my care professional sees how I am doing or might even contact me if my glucose values are worsening.’* By sharing data, the patient's and the professional world are linked.

This might seem an ideal situation, but currently the use of eHealth among patients with T2DM is low and half of our participants did not know about which eHealth tools were available. In addition, care professionals did not know which eHealth tools their patients were using. Also in our study, most participants were dissatisfied with the lack of knowledge of professionals regarding eHealth. Participants stated that there is no interest in eHealth and digitalization is only slowly proceeding in the healthcare system. On the other hand, participants thought it was a logical consequence of the development process. The developments in eHealth and transitions in care are going too fast to keep up with, it has all proliferated quite quickly. *The development is much faster than my general practitioner can anticipate on.’* General practitioners are often unfamiliar with the possibilities of eHealth, and there is no direct link to the electronic health records to track their patients.^2,6^ Also some participants would argue in favour of automatically transferring their self-monitoring data to professionals. They emphasized on some visions of potential care practices and their relationship with a care professional. They imagine that professionals can check the data when needed, and in case of undesired glucose levels the professionals can immediately react and contact their patients, maybe by a system providing an alarm to the professional. This would change the patient–professional relationship and result in personalized care. Although it is often expected by our participants that the patient–professional relationship might diminish with digital health, digital intimacy between professionals and patient is possible.^53,54^

## Discussion

In the present study, we examined whether, how, and under which conditions patients with T2DM integrate eHealth apps in their self-management practices, and how this affects their daily lives and relationships with others. Four different apps were introduced to investigate the lives and self-management practices of patients with T2DM, and the possible roles, added values, and challenges of eHealth in their daily practices. The patients with T2DM involved in this study were asked to choose one of the four apps. Aligning with a number of further practice-based studies on eHealth, it became clear that eHealth apps do not work automatically for everyone, but that it depends a lot on whether users are able to integrate apps and the information these create in a meaningful way into their individual practices and sense-making about themselves and their personal health situation.^9,29,47^ Still, as Pinto et al. highlight, it remains a challenge for patients to find a way to incorporate the use of eHealth in their daily lives.^5^ In particular, personal, individualized health knowledge turned out to be an important topic throughout this research. Participants were particularly interested in using eHealth apps to better understand *their* diabetes, that is, how their body responds to nutrition, physical exercise and how this relates to the types of bodily signals they perceive. Most already perceived that their body responds differently from those of others, and personalized knowledge and data is needed to understand their body well. Interpreting the data is key in giving someone power to take actions.^52^

Some of our participants experienced that the outcome is not always aligned with bodily feelings, which was similar to findings of Smith & Vonthehoff.^55^ However, they found that eHealth users contrast the data with their bodily sensation, and considered the data as more accurate than their embodied mode of knowing.^55^ In our study, the participants did not consider the data as more accurate than their personal sensations. Like Algera,^1^ most of our participants underscored the value of having insight and understanding of their body through data, but feelings and data should be critically compared and questioned as part of someone's personal health knowledge. As our participants explained, the data provide an additional view into their body and illness, could be considered as complimentary to sensations, and associated with different aspects of the illness. Algera suggests that the obtained data could function as a kind of training kit for patients to learn about their body, and the intertwining of eHealth data and bodily sensation could enhance or form a new sensory capacity.^1^ This was as well in line with earlier research^47^ and the perception of our participants to learn, act, and see changes in their weight already in the first months after using the app. Apparently, most patients with T2DM could benefit from the apps as part of self-perception practices and other practices related to nutrition and physical activity. It became a rearrangement of personal, family, and care practices that worked well for them.^56^ It remains a topic for further research, under which conditions users may be inclined to prioritize measurement data over bodily sensation or bodily sensations over data.

While many participants felt quite able to use and make sense of the eHealth apps, some of our participants valued assistance, because they experienced a misalignment between data and bodily sensations causing anxiety, or got obsessed or ‘addicted’ with a tool. Some participants felt overwhelmed or emotionally affected by an encounter with the data or even obsessively depend on the data instead of listening to their bodies. Similar findings of self-tracking becoming rather an emotional and practical burden for users have been shown by Lupton and Sjöström et al.^9,57^ The topic of personal support in use and understanding of eHealth apps and data was another important topic according to our participants. Milne et al. also show that personal assistance is needed, but it is often unclear where someone could go or to whom could they reach out and talk.^48^ This challenge in practice needs to be overcome to create sustainable embedding of eHealth in care of patients with T2DM.^5^

The applied social practice approach was particularly suitable for tracing how people integrate new devices and data into their practices. Our participants pointed towards the role of the care professionals and mentioned that data obtained through eHealth are often not incorporated in current care practices. Besides having the opportunity to receive personal assistance in understanding the data, the use of obtained data through eHealth could also have several benefits for the patient–professional relationship. First, patients with T2DM learn more about diabetes and glucose levels, and how this has a relation with nutrition, physical activity, their diabetes and body. This can support treatment acceptance and adherence and avoid exacerbation of diabetes. Second, sharing of data could improve the relationship due to a more personal focus and additional input for regular consults. However, it is unknown how the care professionals would envision the embedding of these apps in the care practices. In former studies it was found that the professional needs enough time to review the data before meeting with the patient,^58^ and that general practitioners tend to have a positive attitude towards referring patients with T2DM to eHealth.^2,30,58^ However, as our participants observed, the professionals are often unfamiliar with the multiplicity of available apps, and there is no direct link to the electronic health records to share the data. Vonken et al. also explored the unfamiliarity of eHealth for T2DM among Dutch general practitioners,^33^ and Jakobsen et al. showed that a link with the patient record system was an important prerequisite for professionals to accept the use of eHealth as part of their care practice together with their patients.^30^ A further challenge could arise from the strong focus of patients in the individual expression of their disease that may not easily align with an orientation of health professionals on more generalizable forms of health knowledge, a topic that should be explored in future research.

### Strengths and weaknesses

The combination of three different data collection steps with different research methodologies increases the credibility of the data and study. In each step a different group of participants was included, respectively 16, eight, and 25. Table 1 shows the variety of participants regarding age, gender, and disease duration. There is no balanced distribution, but this imbalance is comparable to the Dutch population of patients with T2DM. There are more males suffering from T2DM and the prevalence increases with age.^59^ Furthermore, the demographics of our participants is comparable to other studies in which the average age is above 55 and the average disease duration above 13 years.^60,61^ As discussed by the participants, in an earlier stage of having T2DM, you are less interested in taking strict measures or change your lifestyle. Although probably most participants had a positive stance towards technology, we were able to include non-users of technology in all study parts.

Many of our findings are in line with and confirm earlier research on the use of eHealth by patients with diabetes. Most earlier findings were based on research with patients with T1DM, who are on average younger and thus likely to show a higher affinity with digital technologies and may face slightly different challenges in coping with their disease. Our findings indicate that despite these differences T2DM patients experience the value and challenges of eHealth apps similarly and that some of the challenges, in particular those related to integrating eHealth into regular care practices are a broader and structural issue in the diabetes care system. It furthermore became visible that the specific value of eHealth may not so much lie in providing a one size fits all approach in diabetes treatment, but that exactly the ability of some apps to support the development of individualized health knowledge has been perceived as an important value to the participants of our study. It may, however, well be that precisely this individualized character may create further challenges for integrating some of the eHealth practices with professional care practices that are arguably oriented towards more generalized health knowledge. These challenges surely constitute an important avenue for further research.

Using a social practice perspective provided the focus on self-management and self-perception practices, and how the apps would fit or rearrange practices. Besides barriers and drivers of eHealth,^40,41^ this social practice perspective adds a different layer to the analysis compared to studies fully focussing on use or user experiences. The applied social practice lens highlighted that the change of life without T2DM towards a life with T2DM should not be taken for granted, it requires new practices that become the new ‘normal’. In the case of T2DM, the social practice approach was used to provide an understanding of the changing practices that included relations to and between all aspects in their life with T2DM and the different technologies. Being diagnosed and having T2DM could be seen as a rearrangement of individual, family, and care practices anchored in other practices.^56,62^ In this study these were for example, listening to their bodily sensations and matching these with obtained data, as well as integration of technologies and data in consultations with a care professional or joint activities with family members.

As mentioned before, the participants argue in favour of a more prominent role of healthcare professionals. However, it is unclear how to proceed towards actual use in healthcare and how the practices will and can change. This can cause anxiety and resistance towards uptake of new ‘treatments’ with eHealth. Perspectives of healthcare professionals and other stakeholders need to be investigated. There is seemingly a struggle for patients to use personal heath data, acquired through eHealth, together with their healthcare professionals, but it is unknown where the struggles originate from according to the diabetes care professionals. The social practice perspective could provide additional value by exploring how current diabetes care practices are the status quo and how these change with the embedding of eHealth apps.

Our study design including four different eHealth apps was aimed at creating a broad qualitative understanding of the possible range of uses and effects of eHealth apps for T2DM patients, yet not at a systematic comparison of the benefits and drawbacks of different apps and for different subsets of patients. Such a more systematic comparison of eHealth apps with different functionalities can be a useful direction for future research. Another limitation was the low number of participants with lower digital literacy and a more varied cultural background.^44,63^ The methodological triangulation^43^ and rich data set used to support the finding of this study is a main strength. Data collected and findings of each step supported the method of data collection in the following step, for example, the topics discussed in the focus groups of the second data collection step were based on the findings of the first individual interviews, and the chosen apps as part of the third data collection step were based on the needs expressed by participants of the first and second step.

## Conclusion

After receiving the diagnosis T2DM, patients need to change their daily practices to decrease exacerbations of diabetes. Our study showed that under certain conditions, eHealth can play an important role for patients in developing a nuanced, personalized understanding of their body and cope with T2DM. Coping with diabetes is a learning process in which patients learn to understand and listen to their body. Data obtained through eHealth has the potential to support in sensing their body and altering food and physical activity practices. A prerequisite is that eHealth needs to be fitted into the specific practices of users, and patients desire a strong role by their care professionals in providing support in interpretation of data.

## Notes

1. TOPFIT Citizenlab is a three-year research and innovation programme focussing on *Citizen Science*. In different studies within this programme citizens, healthcare professionals and companies join forces with researchers to develop and implement technological innovations for health and healthcare. More information: www.topfitcitizenlab.nl.

## Appendix 1. Interview guide exploration interviews

This interview guide was prepared as a set of questions to use during the first interviews with users and non-users of eHealth for T2DM. The goal of these interviews was to explore how patients cope with T2DM in their daily life, and their stance towards eHealth apps.

1. Can you introduce yourself?
  - Can you elaborate on your life with type 2 diabetes?
2. Which care professionals assist you in the management of diabetes? How often do you see your care professional and what do you discuss?
3. Do you use apps in your life with diabetes?
  - If yes, which apps do you use because of diabetes? How do you use these apps? How do these apps support you in your life with diabetes?
  - If no, would you like to use apps in your life with diabetes? What do you expect from using an app in your life with diabetes?
4. Do you know any apps that could support you with diabetes?
  - If yes, which apps do you know? If different from previous question: Have you used these apps? How did you experience these apps?
  - If no, are you interested to know more about apps you could use in your life with diabetes?
5. Have you spoken with care professionals about the use of apps for diabetes?
  - Do you need help in using apps? What kind of assistance would you expect from your care professional?
6. Will you share data obtained in the apps?
  - If yes, with whom would you share the data? What is the reason you would share the data?
  - If no, what is your reason for not sharing the data?
7. Which expectations do you have considering the future and your life with diabetes?

What would you wish for the future?

## Appendix 2. Interview guide interviews before using eHealth apps

The interview guide was prepared to start talking about expectations of eHealth as part of life with T2DM. The goal of these interviews was to understand their self-management activities before using the app and their expectations of the app.

1. Can you briefly tell us about yourself, and your life with diabetes?
2. What is the influence of diabetes on your daily life?
  - Have you tried or do you use other eHealth apps for diabetes self-management? If yes, which applications. If no, what is your reason not to use any?
  - Do you already use other eHealth apps for active and healthy lifestyle? If yes, which applications. If no, what is your reason not to use any?
3. What is your motivation to participate in this study?
  - How did you got informed about this study?
4. What is your motivation to start testing eHealth apps for diabetes?
  - Which app did you choose? What is your main reason for choosing this app?
  - What would you like to try with the app?
  - How is your stance towards technology?
5. What are your goals and expectations with this eHealth app?
  - Do you think this app could influence your daily life? If yes, what might change?
  - Do you think this app could help to improve self-management and control of diabetes or help to improve a healthy lifestyle? If yes, how would you imagine this?
6. How is the support you receive from family members or friends? How will they support you in using eHealth to cope with diabetes?
7. Have you received any information on eHealth from your healthcare professional?
  - If yes, which information did you receive?
  - If no, do you think this should be a task of your healthcare professional?
8. How do you imagine the use of eHealth as part of care and your relationship with healthcare professionals?
  - Do you think the care might change with the use of eHealth? If yes, what might change?

## Appendix 3. Topic list focus groups after using eHealth apps

This topic list was prepared to use during the focus groups. The goal of these focus groups was to share experiences after having used the app for a couple of months, how it contributed or changed their self-management activities, and to what extent it is or could be used as part of daily life. The topic list for these focus groups was prepared in collaboration with two participants.

1. Experiences
  - With the eHealth app
  - Which were pleasant or unpleasant
  - Thoughts about advantages and disadvantages
  - Experiences of problems
2. Daily life experiences specific on:
  - Blood glucose levels
  - Physical exercise
  - c. Diet
  - Stress and emotions
3. Actual use
  - Frequency of use (daily/weekly)
  - Involvement of social network
4. Changes towards:
  - Diabetes self-management and control
  - Healthy and/or active lifestyle
5. Intention to use over a longer period of time
6. Healthcare professionals
  - Are they informed by the participant about use of eHealth
  - Reactions of healthcare professionals regarding eHealth, use, and need for more information
7. Desirable and offered support (medical/technical/daily life)
8. Experiences, wishes, and needs for information and education regarding eHealth
